# Extra Surfactant-Assisted Self-Assembly of Highly Ordered Monolayers of BaTiO_3_ Nanocubes at the Air–Water Interface

**DOI:** 10.3390/nano8090739

**Published:** 2018-09-18

**Authors:** Hiroki Itasaka, Ken-Ichi Mimura, Kazumi Kato

**Affiliations:** 1 Inorganic Functional Materials Research Institute, National Institute of Advanced Industrial Science and Technology (AIST), 2266-98 Anagahora, Shimoshidami, Moriyama-ku, Nagoya, Aichi 463-8560, Japan; k.mimura@aist.go.jp; 2 National Institute of Advanced Industrial Science and Technology (AIST), Central 1, 1-1-1 Umezono. Tsukuba, Ibaraki 305-8560, Japan; kzm.kato@aist.go.jp

**Keywords:** self-assembly, evaporation-induced self-assembly, nanocrystal, nanocube, barium titanate, surfactant, oleic acid

## Abstract

Assembly of nanocrystals into ordered two- or three-dimensional arrays is an essential technology to achieve their application in novel functional devices. Among a variety of assembly techniques, evaporation-induced self-assembly (EISA) is one of the prospective approaches because of its simplicity. Although EISA has shown its potential to form highly ordered nanocrystal arrays, the formation of uniform nanocrystal arrays over large areas remains a challenging subject. Here, we introduce a new EISA method and demonstrate the formation of large-scale highly ordered monolayers of barium titanate (BaTiO_3_, BT) nanocubes at the air-water interface. In our method, the addition of an extra surfactant to a water surface assists the EISA of BT nanocubes with a size of 15–20 nm into a highly ordered arrangement. We reveal that the compression pressure exerted by the extra surfactant on BT nanocubes during the solvent evaporation is a key factor in the self-assembly in our method. The BT nanocube monolayers transferred to substrates have sizes up to the millimeter scale and a high out-of-plane crystal orientation, containing almost no microcracks and voids.

## 1. Introduction

Nanocrystals have been attracting interest owing to their size-dependent physical properties and potential applications in novel electric, optical, and magnetic devices [1,2]. Recent progress in solution-based chemical processes has enabled the synthesis of nanocrystals with well-defined shapes (e.g., sphere, cube, cuboid, octahedron, and dodecahedron), narrow size distributions, and various compositions [3,4,5,6,7,8]. The progress in the synthesis of nanocrystals has also accelerated the fundamental studies of their intrinsic and collective properties. In this context, the assembly of nanocrystals into ordered arrays has become increasingly important for their application as well as fundamental studies. Moreover, the assembly techniques should be able to control the arrangement and crystal orientation of nanocrystals over large areas. 

Self-assembly of nanocrystals is a practical way to achieve their highly ordered arrays that are uniform and continuous over large areas. In particular, evaporation-induced self-assembly (EISA) has been intensely employed by many researchers to fabricate nanocrystal arrays because of its simplicity and controllability. The mechanism of this type of self-assembly is generally explained by the combination of convective flows of solvent driven by solvent evaporation and lateral capillary forces. As the solvent of a nanocrystal suspension evaporates, the nanocrystals dispersed in the suspension are carried to the evaporation front by convective flows and assembled into ordered arrays by lateral capillary forces acting between the nanocrystals [9,10]. Interaction between the surfactant-capped surfaces of nanocrystals is also an important factor to determine their arrangement [11]. In this type of self-assembly, nanocrystal arrays are assembled onto a solid surface or liquid surface. Given the practical applications of nanocrystal arrays, the former case can provide a simpler fabrication process than the latter because the latter requires a process of transferring the nanocrystal arrays floating on a liquid surface to a solid surface. For this reason, various attempts have been made to form two- and three-dimensional arrays of nanocrystals with diverse shapes, sizes, and compositions directly on substrates [12,13,14,15,16,17]. We have previously reported the fabrication of highly ordered three-dimensional arrays of perovskite cubic-shaped nanocrystals (called nanocubes) directly on substrates by using EISA methods: a capillary force-assisted self-assembly method and a dip-coating method [18,19,20,21,22,23,24]. Such direct fabrication on a solid surface including our previous cases, however, commonly suffers from the generation of microcracks during solvent evaporation. Microcracks divide nanocrystal arrays into small domains and render the fabrication of continuous and uniform films of nanocrystal arrays over large areas difficult. In fact, the lateral size of the continuous regions in the three-dimensional arrays was limited to less than a few tens of micrometers in our previous methods. This is a serious problem in practical applications of nanocrystal arrays. Fabrication methods using EISA on a liquid surface have the potential to resolve this problem because nanocrystals on a liquid surface are mobile and can rearrange their positions. Although this advantage has been proven in several reports [25,26,27,28] that demonstrated the formation of monolayers and multilayers of nanocrystals over large areas, most examples are limited to spherical nanocrystals. 

In the present study, we demonstrate the formation of large-scale highly ordered monolayers of barium titanate (BaTiO_3_, BT) nanocubes capped with oleic acid on a water surface by introducing a new EISA method. In our method, oleic acid, used as an extra surfactant, is added to a water surface before the BT nanocube suspension is dropped onto the surface. We found that the addition of the extra surfactant allows the EISA of BT nanocubes to arrange into highly ordered monolayers under the condition in which monolayers do not form without an extra surfactant. Although excess ligands added to a nanocrystal suspension or a nanocrystal-dispersed water surface promote the formation of nanocrystal arrays and improve their ordering by slowing the solvent evaporation or by modifying the interaction between the nanocrystals [12,29,30], we experimentally confirmed that the major role of the extra surfactant in our self-assembly method is distinguished from that of such excess ligands. Our measurements of surface pressure revealed that the extra surfactant exerts compression pressure on the BT nanocubes on the water surface. This compression promotes the EISA of BT nanocubes and prevents the generation of microcracks and voids, resulting in the formation of uniform monolayers with a size of up to a few millimeters. 

## 2. Materials and Methods

BT nanocubes were synthesized by a hydrothermal method. The details of the fabrication process have been mentioned in our previous report [7]. Titanium(IV) bis(ammonium lactate) dihydroxide (Sigma Aldrich Japan, Tokyo, Japan) and Ba(OH)_2_·8H_2_O (FUJIFILM Wako Pure Chemical Co., Osaka, Japan) were used as starting materials of Ti and Ba, respectively. They were dissolved in distilled water and mixed with NaOH (FUJIFILM Wako Pure Chemical Co., Osaka, Japan), *tert*-butylamin (Sigma Aldrich Japan, Tokyo, Japan), and oleic acid (FUJIFILM Wako Pure Chemical Co., Osaka, Japan). The aqueous solution was heated at 220 °C in an autoclave for 72 h. The BT nanocubes synthesized via this process were capped by oleic acid and enclosed by {100} facets [7]. Their typical size was in the range of 15–20 nm. The BT nanocubes obtained were rinsed with ethanol and dispersed in mesitylene. 

**Figure 1:**
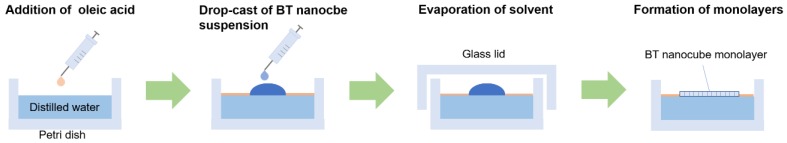
Schematic illustration of our self-assembly process. First, oleic acid diluted with toluene is added to a water surface in a petri dish. After toluene evaporation, the barium titanate (BT) nanocube suspension (mesitylene solvent) is dropped onto the oleic acid-added water surface. The petri dish is covered by a glass lid during mesitylene evaporation under room temperature. As a result of mesitylene evaporation, BT nanocube monolayers form at the air-water interface.

Figure 1 shows a schematic illustration of our self-assembly method. Distilled water was poured into a petri dish (7.0 cm in diameter), and the water surface was cleaned using an aspirator. Oleic acid was diluted with toluene to a concentration of 1.9 × 10^−3^ M, and the solution was added to the water surface with a microsyringe. Toluene was evaporated at room temperature so that oleic acid molecules disperse over the water surface. After toluene evaporation, 40 µL of the BT nanocube suspension was dropped onto the oleic acid-added water surface with a microsyringe. The petri dish was covered with a glass lid to slow the evaporation of mesitylene under room temperature. The resulting monolayers of BT nanocubes floating on the water surface were transferred to silicon substrates by the Langmuir-Schaefer deposition method. The silicon substrates were subjected to only ultrasonication in ethanol before monolayer deposition to keep their surface hydrophobic. After monolayer deposition, the substrates were dried at 60 °C followed by ultraviolet light irradiation for 2 h and drying at 200 °C for 1.5 h to remove organic residues. 

The morphologies of BT nanocube monolayers on substrates were observed using a field-emission scanning electron microscope (FE-SEM; JSM-6335FM, JEOL, Tokyo, Japan). The out-of-plane orientation of BT monolayers on substrates was evaluated by performing X-ray diffraction (XRD) measurements using a SmartLab XRD (Rigaku, Tokyo, Japan) with a Cu Kα radiation source.

The surface pressure on the water subphase surface was measured by the Wilhelmy plate method with a KSV NIMA Layer Builder (Biolin Scientific, Espoo, Finland) to investigate the dependence of surface pressure on the amount of oleic acid added to the water surface. Additionally, real time measurements of surface pressure on the water surface during the solvent evaporation were performed. In the measurements, the process of formation of BT nanocube monolayers was the same as mentioned above; however, the petri dish and the instrument were covered with a plastic case during mesitylene evaporation instead of covering with a glass lid.

## 3. Results and Discussion

**Figure 2:**
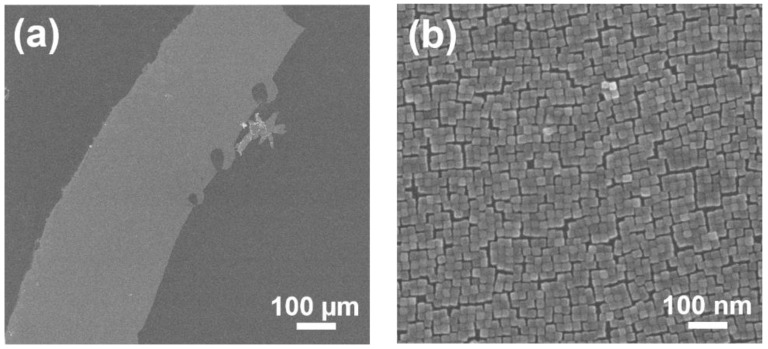
Scanning electron microscope (SEM) images of BT nanocube monolayer transferred on a silicon substrate. (**a**) Entire image and (**b**) magnified image of the monolayer.

In a typical procedure for the formation of BT nanocube monolayers, 40 µL of the oleic acid solution (corresponding to the amount of oleic acid molecules, 2.0 × 10^−9^ mol/cm^2^) is added to the water surface, and then, 40 µL of BT nanocube suspension with a concentration of about 0.2 mg/mL is dropped onto the surface. The BT nanocube monolayers obtained via the typical procedure are a few hundreds of micrometers to a few millimeters in size. We employed the Langmuir-Schaefer deposition method since it is suitable for transferring locally floating monolayers on a water surface to a substrate and has been used in similar systems [25,27]. Figure 2a shows an example of a BT nanocube monolayer transferred to a silicon substrate. It can be seen that the monolayer (the bright region in Figure 2a) is uniform and contains no microcracks over the entire area. The magnified image in Figure 2b shows that the BT nanocubes are highly ordered and form small domains with about 10 nm gaps in the monolayer. Since the thickness of the oleic acid-capped layers of BT nanocubes is about 1 nm [31], these gaps seem to be caused by slight variations in the size and shape of the BT nanocubes, and by the penetration of extra oleic acid molecules between the neighboring BT nanocubes. Although we also obtained BT nanocube multilayers by using a more concentrated suspension (more than 2.0 mg/mL), we will focus on the formation of monolayers in this study.

**Figure 3:**
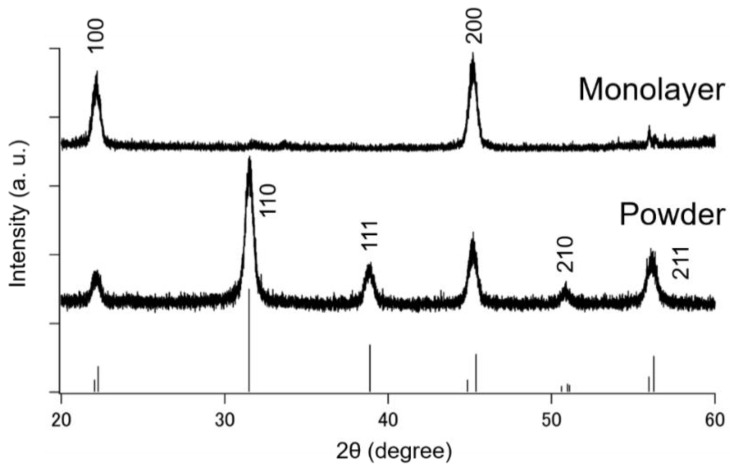
X-ray diffraction (XRD) patterns of BT nanocube monolayer with an area of about 0.3 cm^2^ on a silicon substrate and BT nanocube powder. The vertical lines represent the diffraction pattern of tetragonal BT (P4mm).

The crystal orientation of the BT nanocube monolayer obtained was analyzed from its XRD pattern. Figure 3 shows the results of XRD 2θ-ω scan of the BT nanocube powders and a BT nanocube monolayer with an area of about 0.3 cm^2^ on a silicon substrate (1 × 1 cm^2^). Although the splitting of (100), (200), (210), and (211) diffraction peaks cannot be observed due to peak broadening caused by their nanometric size, the diffraction pattern of the BT nanocube powder is roughly consistent with that of the tetragonal BT (P4mm) represented by the vertical lines in Figure 3. On the other hand, the diffraction pattern of the monolayer shows only (100) and (200) peaks at 22° and 45°, respectively. This result clearly indicates that the BT nanocubes in the monolayer have an excellent {100} out-of-plane orientation over the large area. The weak diffraction peaks around 56° in the diffraction pattern of the monolayer were proved to be from the Si substrate, as determined by the XRD measurement of the substrate without BT nanocube monolayers.

**Figure 4:**
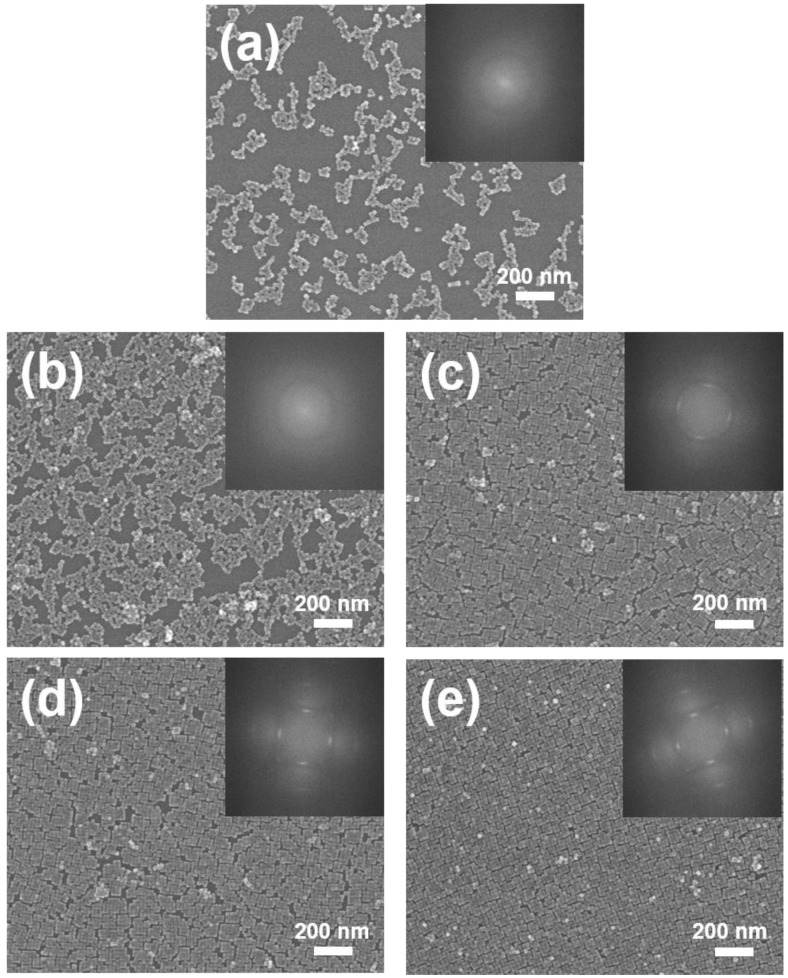
SEM images of BT nanocubes transferred to silicon substrates from water surfaces with the addition of various amounts of oleic acid: (**a**) 0; (**b**) 2.5 × 10^−10^; (**c**) 5.0 × 10^−10^; (**d**) 1.0 × 10^−9^, and (**e**) 2.0 × 10^−9^ mol/cm^2^. The insets show the fast Fourier transform (FFT) patterns of the SEM images.

To investigate how the amount of extra oleic acid influences the self-assembly of BT nanocubes, we performed the self-assembly process by changing the amount of oleic acid from 0 to 2.0 mol/cm^2^. Figure 4 shows the SEM images of BT nanocubes transferred to silicon substrates from the water surfaces with the addition of various amounts of oleic acid. These images demonstrate that the amount of oleic acid strongly affects the ordering of BT nanocubes. Without the addition of oleic acid (0 mol/cm^2^), two-dimensional random clusters consisting of a few tens of BT nanocubes were deposited on the substrate; however, their monolayers were not observed (Figure 4a). This may be due to the high boiling point of mesitylene (165 °C), which causes slow solvent evaporation at room temperature. Under the condition, BT nanocubes gathered at the evaporation front by convective flows, dispersing away from the suspension droplet before being assembled into monolayers by lateral capillary forces, because the supply rate of BT nanocubes to the evaporation front is not enough for the formation of monolayers. The addition of 2.5 × 10^−10^ mol/cm^2^ of oleic acid caused the formation of BT nanocube monolayers with a size of a few micrometers. However, the monolayers formed in this condition contain numerous voids and have poor ordering of BT nanocubes, as shown in Figure 4b. When more than 5.0 × 10^−10^ mol/cm^2^ oleic acid was added, BT nanocube monolayers grew to more than submillimeter sizes, and voids were dramatically reduced (Figure 4c–e). Moreover, the fast Fourier transform (FFT) patterns of the SEM images shown in the insets of Figure 4 clearly demonstrate an improvement in BT nanocube ordering with increasing amount of oleic acid. A four-fold symmetry, which indicates the long-range order of BT nanocubes in the monolayers, appears in the FFT patterns when the amount of oleic acid is more than 5.0 × 10^−10^ mol/cm^2^ (the insets in Figure 4c–e), while no symmetry can be observed at oleic acid contents of less than 2.5 × 10^−10^ mol/cm^2^ (the insets in Figure 4a,b). These results revealed that the addition of extra oleic acid allows BT nanocubes to form large-scale monolayers and increasing the amount of extra oleic acid improves the ordering of BT nanocubes. 

**Figure 5:**
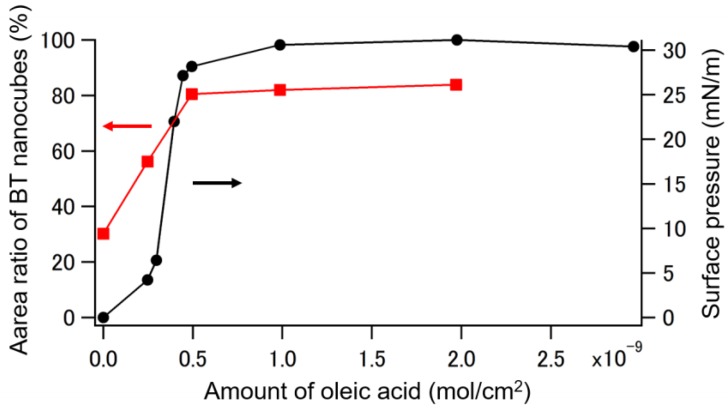
Surface pressure on the water subphase surface (black filled circles and solid line) and mean area ratio of BT nanocube in a 1.6 μm × 1.9 μm area on the substrate (red filled squares and solid line) plotted against the amount of oleic acid added to the water surface.

To further understand the effect of the amount of oleic acid, we measured the surface pressure on the water subphase surface on which various amounts of oleic acid were added but without the drop-cast of the BT nanocube suspension. The measured values were plotted as a function of the amount of oleic acid (black filled circles and solid line in Figure 5). The surface pressure saturates to around 30 mN/m when the amount of oleic acid exceeds about 5.0 × 10^−10^ mol/cm^2^. This saturated value is well consistent with the equilibrium spreading pressure of a monolayer of oleic acid molecules on a water surface [32]. Interestingly, both the appearance of the four-fold symmetry in the FFT pattern (Figure 4) and the saturation of surface pressure were observed when the added amount of oleic acid exceeded 5.0 × 10^−10^ mol/cm^2^. This concurrence may indicate that the ordering of BT nanocubes in their monolayers is related to the surface pressure of the water surface. We evaluated the density of BT nanocubes quantitatively by calculating their area ratio on the substrate from the SEM images using the image processing software, ImageJ. We calculated area ratios in five randomly selected regions (1.6 μm × 1.9 μm in area) covered by BT nanocubes for each sample. The mean values of area ratio for each sample are plotted in Figure 5 as a function of the amount of oleic acid (red filled squares and solid line). The plots also indicate the dependence of the area ratio on the surface pressure. The area ratio increases with an increase in surface pressure and saturated with the saturation of surface pressure. Therefore, these results indicate that the surface pressure exerted by the extra oleic acid is a key factor in the formation of BT nanocube monolayers and the arrangement of BT nanocubes in our method. 

**Figure 6:**
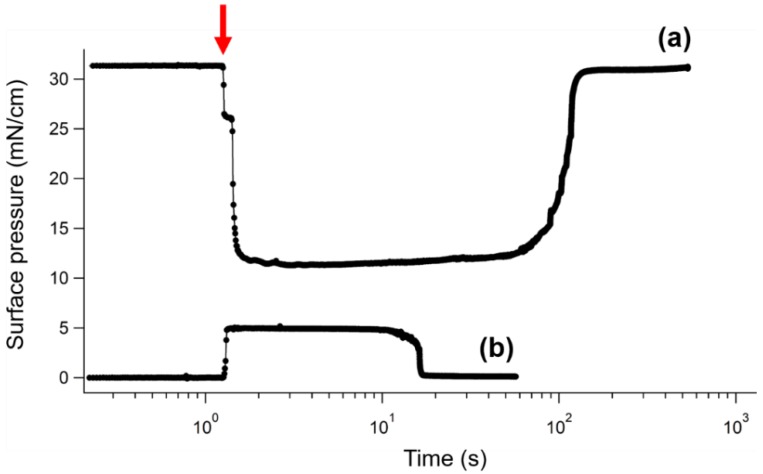
Time profiles of surface pressure on water surfaces (**a**) with the addition of 2.0 × 10^−9^ mol/cm^2^ of oleic acid and (**b**) without oleic acid. The red arrow indicates the time at which BT nanocube suspension was dropped onto the water surface.

Since the aforementioned measurements of surface pressure were performed in the absence of BT nanocubes and their suspension, it remains unclear how the extra oleic acid affects the surface pressure during the assembly of BT nanocubes. It should be noted that the compression of the suspension droplet by oleic acid could be visually confirmed from the behavior of the suspension droplet on the water surface. The suspension droplet on the oleic acid-added water surface maintains a lens-like shape and floats on the center of the surface during evaporation, while the suspension dropped onto the water surface without oleic acid immediately spreads out to form thin films and covers the water surface. To investigate this point quantitatively, we measured the surface pressure on the water surface with and without the addition of oleic acid during solvent evaporation in real time. Figure 6a,b show the time profiles of surface pressure on the water surfaces with 2.0 × 10^−9^ mol/cm^2^ of oleic acid and without oleic acid, respectively. It is noteworthy that the surface pressure on the oleic acid-added water surface is higher than that on the water surface without oleic acid throughout the measured time range, although these profiles show the different behaviors of surface pressure in response to the drop cast of BT nanocube suspension (indicated by the red arrow) and the evaporation of the solvent. This verifies that extra oleic acid exerts a compression pressure on the suspension droplet and BT nanocubes. The surface pressure of the oleic acid-added water surface decreased when the BT nanocube suspension was dropped and increased to its initial value before the addition upon solvent evaporation. The decrease in surface pressure may be explained by the dissolution of a part of oleic acid molecules on the water surface in the suspension. As the solvent evaporates, the oleic acid molecules in the suspension were released to the water surface and increased the surface pressure. Contrarily, on the water surface without oleic acid, the surface pressure increased in response to the drop cast of the suspension and then decreased to nearly 0 mN/m after solvent evaporation. The increase in surface pressure can be attributed to the presence of suspension spreading over the water surface. The longer evaporation time for the oleic acid-added water surface than that for the water surface without oleic acid may be due to the difference in the behavior of the suspension droplet on the water surfaces, which causes a difference in the specific surface area of the suspension droplet. The oleic acid molecules dissolved in the suspension may also contribute to the slowing of evaporation. The slow evaporation of solvent is favorable to the formation of uniform monolayers.

**Figure 7:**
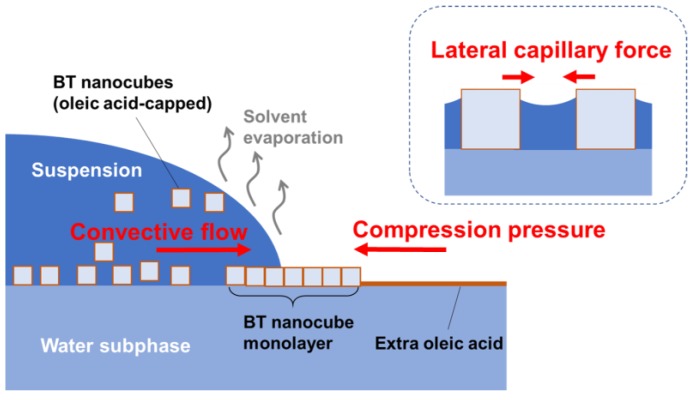
Schematic illustration of mechanism of oleic acid-assisted self-assembly of BT nanocubes. The extra oleic acid added to the water surface exerts a compression pressure on BT nanocubes, which are carried to the evaporation front by convective flows. This compression pressure confines the BT nanocubes around the suspension droplet and assists the lateral capillary forces to work between the BT nanocubes.

From the results mentioned above, we concluded that the major role of extra oleic acid in our method is as follows: the extra oleic acid added to the water surface exerts a compression pressure on the BT nanocubes, which were carried to the evaporation front by convective flows, as illustrated in Figure 7. This compression pressure confines the BT nanocubes around the suspension droplet and assists the lateral capillary force to work between the BT nanocubes, leading to the formation of a highly ordered arrangement of BT nanocubes. Furthermore, it can be deduced that the compression by extra oleic acid reduces the generation of microcracks. 

**Figure 8:**
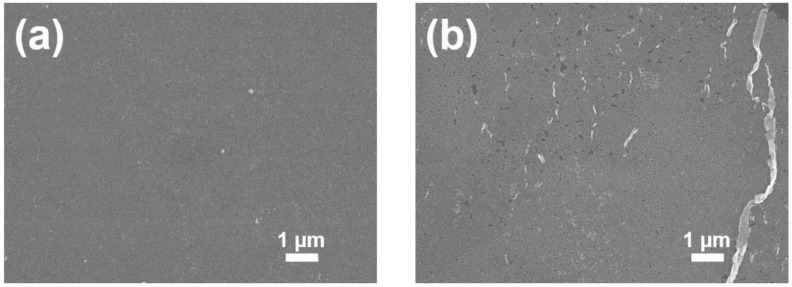
SEM images of BT nanocube monolayers fabricated using (**a**) our method and (**b**) Langmuir–Blodgett method.

Finally, we compared the quality of BT nanocube monolayers fabricated using our method and the Langmuir-Blodgett (LB) method in which nanocrystals on a liquid surface are assembled into monolayers by compressing them using movable barriers. The LB method is commonly used for fabrication of nanocrystal monolayers [33,34]. Since the LB method is similar to our method in terms of using compression pressure for fabrication of monolayers on liquid subphases, this comparison seems helpful in evaluating the quality of monolayers produced by our method. In this experiment, the LB method was carried out using the same instrument that was used for the measurement of surface pressure. BT nanocubes, which were dispersed on the surface of distilled water poured into a Teflon Langmuir trough, were compressed by movable barriers with a compression pressure of about 50 mN/m. The compression pressure was selected to be slightly lower than the collapse pressure of the monolayer (about 55 mN/m). Silicon substrates used in the LB method were subjected to ultraviolet rradiation to make their surface hydrophilic. Our method was carried out under the same condition as that used for the fabrication of the monolayer shown in Figure 2. Figure 8a,b show the SEM images of BT nanocube monolayers fabricated using our method and the LB method, respectively. In Figure 8a, almost no microcracks and voids exist; however, only a few bright spots, where small two-dimensional clusters of BT nanocubes are deposited on the monolayer, are observed. On the other hand, in Figure 8b, numerous voids are present even though a higher compression pressure was applied. Additionally, some overlaps of monolayers can also be observed as bright areas in the image. Such voids and overlaps are a common problem in the LB method and there is the trade-off relation between them. Increasing the compression pressure to reduce voids should cause overlapping of monolayers, while voids cannot be reduced under a low compression pressure [35]. Thus, severe control of experimental parameters, such as compression pressure, is required to reduce the voids and overlaps. Although the differences in the deposition methods and surface conditions of the substrates can contribute to the resultant morphology of the monolayers, this result seems to demonstrate qualitatively the potential of our method to provide high-quality monolayers without any dedicated instrument and severe control of experimental parameters.

## 4. Conclusions

We demonstrated a novel self-assembly method to fabricate large-scale highly ordered monolayers of nanocrystals. BT nanocube monolayers with a size more than submicrometer scale were achieved by adding an extra oleic acid onto the water subphase surface. SEM observations and XRD measurements revealed that the monolayers obtained contain almost no microcracks and voids, and have a high crystal orientation. The measurements of surface pressure on the water surface revealed that the addition of extra oleic acid assists the EISA of BT nanocubes into a four-fold symmetrical arrangement by exerting a compression pressure on the BT nanocubes. The combination of compression pressure with the EISA allows the formation of large-scale monolayers under the condition where monolayers cannot form only by EISA. Since our method is potentially applicable to other types of nanocrystals, we believe that the present work will open a route to constructing highly ordered and large-scale arrays of various nanocrystals.
